# Homœopathy

**Published:** 1852-03

**Authors:** 


*Homœopathy; an Examination of its Doctrines and Evidences.* By WORTHINGTON HOOKER, M. D.

Doctor Hooker, herein, has done good service to the public, both medical and general. It is really a capital book. He seems to possess just the qualifications necessary for such a work. With a mind clear and scrutinizing, yet fair and candid, with evidently high views of the true scope and mission of medical science, and the medical profession, and in a style of great simplicity and purity, and even elegance, he has produced a book, that, once taken up, it is hard to put down again; and harder still to gainsay or controvert. With a well proportioned admixture of argument, illustration, and playful humor, he makes such an exposition of the singular compound of impudence, folly, and trickery, *Homœopathy*, as must save any unprejudiced mind — any lover of the *true* either in science or morals, from yielding credence to its ridiculous pretensions. As for those who have already unwarily surrendered themselves to favor its shallow plausibilities, especially if from education and reflection they may be supposed to know what a great reality of long and patient observation, and rigid induction, true science is, it is difficult to see how they can save their faces from a slight tingle of mortification at the fatuity they have manifested by such surrender.

The truth is obvious, that not one in a thousand of those who have been and are willing to lend themselves to favor it, have ever given a thorough examination of its pretended facts, and its specious reasonings. As the author well says, "A wordy and fine spun theory, built upon the loosest analogies, especially if accompanied, as is usual with all forms of delusion and quackery, with reports of wonderful cures, is sufficient to satisfy them."

The man of science, and the man of plain common sense, have only need to fairly and closely examine it — in other words, to *sound* it — in order to discover it wofully lacks the ring of true metal.

Our author devotes two chapters to an "Exposition of the System of Hahnemann." These commend themselves to the sense of justice and fair-dealing of the reader by containing quotations at length from their standard authors — giving their own statements of what their doctrines are.

The next two chapters are devoted to an "Examination" of these doctrines. In these, and the succeeding chap. (V), *Homœopathy* is put upon its trial. Herein, he shows up its gross yet cool assumption of ungrown facts, its hair-brained deductions, and its loose analogies. He abundantly convicts it of being inconsistent with well known and fully attested fruths, the logical deductions of scientific observation — inconsistent with reason and common sense, and moreover, inconsistent with *itself.* As a specimen of the latter, out of a multitude as good, or better, we give an extract. It is in reference to the "almost innumerable gradations of impressibility" upon which they base the necessity of varying from the strength of the 'Mother Tincture' to the *decillionth* of a drop.

We quote: If medicines produce in infinitesimal doses "such effects as are attributed to them, and if there be such wide differences in the susceptibility of the sick, it must be very important to fix upon exactly the right dose in each case. And if an infinitesimal dose of a medicine carefully prepared with just the right amount of agitation and trituration, be appropriate to a case, then it would certainly be very injurious to the patient to give a million of such doses at once.

"Nothing can be more obvious; and yet Homœopathists do not appear to be aware of it, for in their dosing of the sick, they jump about among the millionths, billionths, quadrillionths, and decillionths, with a sort of frisky freedom.

"The range of doses in 'Alopathy' is somewhat smaller than the range of doses in Homœopathic practice. For example, while the 'Alopathic' physician calls one-sixth of a grain of tartar emetic a small dose, and three grains (eighteen times that amount) a large one, the Homœopathic calls the decillionth of a grain a small dose, and a million, billion, or quadrillion of such doses; what? why a *small* dose too. The arithmetic of Homœopathy seems to deprive those who venture its airy flights of all power of appreciating differences in quantity. Differences as wide as that between an atom and a world they seem hardly to note or to know.

"That my readers may see that I am not misrepresenting Homœopathic practice, I will refer them to some cases reported by Prof. Henderson. He is perhaps less adventurous than most Homœopathists in his leaps among the millions, and trillions, and decillions, and yet these cases show that it is not at all uncommon for him to change the medicine which he is giving in any case, to the amount of *six*, *twelve*, or even *eighteen* dilutions.

"As one reads these reports of cases, the changes do not strike him as being so very great because they are announced with such small figures. But if he undertakes to estimate them, he finds that they are immense. Thus when Belladonna 12 is changed for Bellad. 6, the alteration seems small, because the *figures* are so. But in reality the drop of Belladonna 6 (the 6th dilution) contains, as I reckon it, just one hundred millions of millions more of the *Bellad.* than a drop of the 12th dilution does. But he makes much greater leaps than these in his dosing."

Think of the consistency of dwelling upon the innumerable gradations of impressibility of patients, and yet making such a terrible leap from a dose of a given power, to one uncounted millions more powerful, with impunity!

In his estimate of Hahnemann, (chap. 6th) occurs the following, which displays our author in a good light. We quote: "One of the peculiarities in the workings of Hahnemann's mind is very remarkable. I refer to his coming to the most stupendous conclusions, without seeming to note, or even to know, when or how he did it. Most discoverers of great truths tell us, and very minutely, by what processes of observation and reasoning they made their discoveries. But *Hahnemann* announces what he claims to be discoveries, and those too of the most astounding character; and yet he informs us of the manner in which his mind was led on to its conclusion, only in relation to a single one of them, viz., the doctrine of the '*Similia Similibus Curantur*.' The doctrine of the efficacy of infinitesimal doses, which if true, is one of the most wonderful truths which was ever discovered, was first announced, as I have already stated, in a note, and that only incidentally; and we are no where informed at what time, and under what circumstances, the 'discovery' was made. One would suppose that a discovery which makes a grain of any medicine sufficient to supply all the inhabitants of the earth centuries, nay, ages upon ages, with all the doses which they would need of that article, would have a date in the mind of its discoverer, and would be reckoned as an era in medicine; and that the circumstances which led to its discovery would be minutely detailed in every notice of it. But no. So stilly did the mountain-mind of *Hahnemann* bring forth this '*ridiculus mus*,' as it has shown itself to be, that no record seems to have been made of the period of its birth."

We are tempted to promote the sale of the work by giving one more extract from the concluding observations on a point, that it strikes us, is eminently worthy of remark. We quote: "The true position of the advocates of Homœopathy should be understood. They attack both the *science* and the profession of medicine. Softly and scientific as are their pretensions, their spirit is the very spirit of radicalism. They aim, as do the advocates of other exclusive and absurd systems less refined and elaborate than this, to destroy the medical profession, and to substitute in its place a mere sect bound together by an ephemeral folly, and founded by one who began his career as an open and unblushing quack.

"In view of the above considerations, we ask the intelligent and influential in the community, to decide whether they will consent to encourage this radicalism in medicine, or whether they will unite in throwing around our profession all those safeguards which are needed to secure its advancement, and to enable it to deliver society from the evils of quackery. The issue is distinct and clear. Every man's influence is thrown into one scale or the other. It is not a light thing that a man does, who gives his countenance to delusion and quackery, even though it be but a momentary act, and an exception to his ordinary treatment of the medical profession. He lends, by this act, his sanction to the whole system of imposture, which the opposers of a well educated profession from *Hahnemann* down to the most ignorant of village quacks, or the basest seller of patent nostrums, are endeavoring to foist upon the community."

But we must refrain from further quotations. We fear we have not done the book justice by the extracts here made. Every page of the work seem to *tell* effectively against the rare combination of flimsy reasonings, loose analogies, assumption, and absurdity, against which it is directed. Doctor Hooker has made it a *work of demolition* in the estimation of every unprejudiced mind that will *read* and *weigh* it. We hope the book will meet with an extensive sale, especially among those who have a lingering doubt of the utter baselessness of the *Rosewater* medicine, upon which it treats.

In his introduction the author says: "I have no desire to search out its *weak* points, and leave untouched its *strong* ones, if there be any. But I am willing to meet it at *every* point."

We think the author has verified this to the letter. P. H. S.

